# Upregulation of Neurotrophic Factors Selectively in Frontal Cortex in Response to Olfactory Discrimination Learning

**DOI:** 10.1155/2007/13427

**Published:** 2007-05-30

**Authors:** Ari Naimark, Edi Barkai, Michael A. Matar, Zeev Kaplan, Nitzan Kozlovsky, Hagit Cohen

**Affiliations:** ^1^Anxiety and Stress Research Unit, Ministry of Health Mental Health Center, Faculty of Health Sciences, Ben-Gurion University of the Negev, Beer Sheva 84170, Israel; ^2^Faculty of Science and Science Education, Brain and Behavior Research Center, University of Haifa, Haifa 31905, Israel

## Abstract

We have previously shown that olfactory discrimination learning is accompanied by several forms of long-term enhancement in synaptic connections between layer II pyramidal neurons selectively in the piriform cortex. This study sought to examine whether the previously demonstrated olfactory-learning-task-induced modifications are preceded by suitable changes in the expression of mRNA for neurotrophic factors and in which brain areas this occurs. Rats were trained to discriminate positive cues in pair of odors for a water reward. The relationship between the learning task and local levels of mRNA for brain-derived neurotrophic factor, tyrosine kinase B, nerve growth factor, and neurotrophin-3 in the frontal cortex, hippocampal subregions, and other regions were assessed 24 hours post olfactory learning. The olfactory discrimination learning activated production of endogenous neurotrophic factors and induced their signal transduction in the frontal cortex, but not in other brain areas. These findings suggest that different brain areas may be preferentially involved in different learning/memory tasks.

## 1. INTRODUCTION

Brain-derived neurotrophic factor (BDNF), a member of the neurotrophin (NT) family of survival-promoting molecules, plays an important role in the growth, development, maintenance, and function of several neuronal systems [1]. It is known to modulate synaptic plasticity and neurotransmitter release in a variety of neurotransmitter systems, as well as intracellular signal transduction pathways [1]. It regulates axonal and dendritic branching and remodeling [2–5], synaptogenesis in arborizing axon terminals, efficacy of synaptic transmission, and the functional maturation of excitatory and inhibitory synapses [6–8].

Activity-dependent synaptic long-term potentiation (LTP); that is, the transcription-dependent electrophysiological correlate of long-term memory [9, 10], is considered a pivotal cellular mechanism underlying learning and memory in which BDNF and TrkB, a protein-tyrosine kinase receptor for BDNF, are involved. BDNF gene deletion or inhibition induces a deficit in learning and memory [9], whereas learning and memory significantly increase circulating and brain levels of nerve growth factor (NGF) and BDNF [11, 12].

It has previously been shown that olfactory discrimination learning elicits several forms of long-term enhancement in synaptic connections between layer II pyramidal neurons in the piriform cortex [13]. Reduced paired-pulse facilitation (PPF) indicates that synaptic release is enhanced [14], while postsynaptic enhancement of synaptic transmission is indicated by reduced rise time of postsynaptic potentials (PSPs) [15] and the formation of new synaptic connections is indicated by increased spine density along dendrites of these neurons [16, 17], while the single spine volume is considerably decreased [18]. Such learning-induced synaptic enhancement occurs three days after olfactory discrimination learning and lasts for several days [14–16]. The mechanisms by which such synaptic modifications are induced are yet to be explored.

Since neurotrophic factors (NFs) appear to be integrally involved in synaptic modification, they presumably precede the physically observable architectural and/or electrophysiological neuronal changes.

This study sought to examine the learning-induced modifications in the expression of mRNA for NFs and compare them to prior findings indicating subsequent olfactorylearning-induced modifications in single neurons in the frontal cortex (FC). The relationship between behavioral training and local levels of mRNA for BDNF, TrkB, nerve growth factor (NGF), and neurotrophin-3 (NT-3) in the frontal and piriform cortex, hippocampal subregions, olfactory bulb, and hypothalamus was assessed 24 hours after olfactory learning. The rationale for this study was that the electrophysiological findings in the FC ought to be preceded by suitable changes in expression of NFs.

The working hypothesis was that olfactory discrimination learning would bring about changes in the expression of mRNA for NF in the hippocampus, as is usual for memory and learning.

## 2. METHODS

All procedures were carried out under strict compliance with the ethical principles and guidelines of the NIH Guide for the Care and Use of Laboratory Animals. All treatment and testing procedures were approved by the Animal Care Committee of the Ben-Gurion University of the Negev, Israel.

### 2.1. Animals

Thirty-six adult male Sprague-Dawley rats (150–200g) supplied by Harlan Laboratories, Jerusalem, Israel, were maintained for the entire duration of the experiment on a 12-hour light-dark cycle with lights on at 7 a.m., room temperature 22 ± 2°C, housed four rats per cage (35 × 60 × 18 cm) on sawdust bedding and provided with water and solid food pellets (Teklad Global Diet 2018S, Harlan Teklad Ltd.,Wis, USA) *adlibitum* . Following a period of habituation to the *vivarium* for 7 days, rats were handled periodically. All stress procedures and tests were performed during the dark phase under dim illumination.

### 2.2. Behavioral paradigms

The rats were randomly assigned into 3 training groups: a trained group, a pseudotrained group, and a naïve group. All were subsequently sacrificed for measurement of NF mRNA levels in dissected brain areas.

#### 2.2.1. Olfactory training

**Figure 1:**
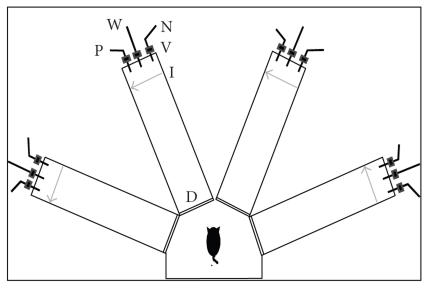
*Schematic description of the olfactory maze*. Protocols for trained and pseudotrained rats are similar: an electronic “start” command randomly opens two out of eight valves (V), releasing a positive-cue odor (P) into one of the arms and a negative-cue odor (N) into another. Eight seconds later, the two corresponding guillotine doors (D) are lifted to allow the rat to enter the selected arms. Upon reaching the far end of an arm (90 cm long), the rat body interrupts an infrared beam (I, arrow) and a drop of drinking water is released from a water hose (W) into a small drinking well (for trained rats, only if the arm contains the positive-cue odor; for pseudotrained rats, in random arms). A trial ends when the rat interrupts a beam, or after 10 seconds, if no beam is interrupted. A fan is operated for 15 seconds between trials, to remove odors.

Prior to training, rats were maintained on a 23.5-hour waterdeprivation schedule. The olfactory discrimination training protocol was performed daily on each trained and pseudotrained rat in a 4-arm radial maze, as previously described by Saar et al. [14–19], with commercial odors that are regularly used in the cosmetics and food industries (Figure 1). Olfactory training consisted of 20 trials per day for each rat [19]. In each trial, the rat had to choose between two odors (positive and negative cues) presented simultaneously. Rats designated to the trained group were rewarded with drinking water upon choosing the positive cue. Rats in the pseudotrained group were rewarded in a random fashion upon choosing any odor. At least 80% of positive-cue choices in the last 10 trials of a training day was defined as the criterion for learning, as was previously used by Staubli et al. [20, 21] and Saar et al. [14–19]. Rats in the naïve group were waterdeprived, but not exposed to the maze.

### 2.3. Processing of brains

Animals were sacrificed 24 hours after olfactory training, with a guillotine in a separate room from the one containing the olfactory learning. Care was taken to avoid additional stress: the area was cleaned between each sacrifice and bodies removed. The brain, brain stem, and cervical spinal cord were removed and the frontal cortex (FC), piriform cortex, hypothalamus, olfactory bulb, and the dentate gyrus (DG), cornu ammonis 1 (CA1), and CA3 subregions of the hippocampus were dissected separately for biochemical studies (Brain Atlas). Samples used were kept at −70°C until the measurements were performed.

### 2.4. Neurotrophic factorm RNA analysis

Brain tissues were brought to room temperature, sonicated for 15 seconds at 50% capacity (ultrasonic processor, Sonic Vibracell TM) and total RNA was isolated using trizol reagent (Molecular Research Center, Cincinnati, Ohio, USA), according to manufacturer's instructions. RNA concentration and purity was quantified according to absorbance at 260nm and 280nm (GeneQuant II, Pharmacia Biothech). RNA was reverse-transcribed into cDNA with Reverse-iT 1st Strand Synthesis Kit (AB-gene, Surrey, UK) for 45 minutes at 42°C in a final 20*μ*L reaction volume containing 1*μ*g of total cellular RNA. To obtain PCR results within the linear range of detection, the cDNA products were diluted 1:40 for BDNF and Trk B, and 1:1000 for *β*-actin (an internal standard for the house-keeping gene). In order to amplify gene-specific sequences, PCR techniques were applied using ReadyMix PCR Master Mix (AB-genes) with specific primer sequences designed from rat mRNA sequences; *BDNF*: *up*-5′*TGGCTGACACTTTTGAGCAC* 3′, *low*-5′ *GCAGTCTTTTTATCTGCCGC* 3′ (genbank accession no. NM-012513), *TrkB*: *up*-5′ *ACTACACCCTGATGGCCAAG* 3′, *low*-5′ *TTGAGCAGGAGCAACATCAC* 3′ (genbank accession no. NM-008745), *NGF*: *up*-5′ *CTGTGGACCCCAGACTGTTT* 3′, *low*-5′ *ATCTCCAACCCACACACACTGAC* 3′ (genbank accession no. NM-013609), *NT-3*: *up*-5′ *TGCAACGGACACAGAGCTAC* 3′, low-5′ *GTGTTTGTCATCAATCCCCC* 3′ (genbank accession no. NM-008742). *β-actin*: *up*-5′ *CTCTTCCAGCCTTCCTTCCT* 3′, *low*-5′ *TAGAGCCACCAATCCACACA* 3′ (genbank accession no. NM-012513). These sequences correspond to nucleotides 254–545 for BDNF, 1073–1261 for TrkB, 510–733 for NGF, 258–639 for NT-3, and 68–320 for *β*-actin. Detection of RTPCR was performed using the T-gradient thermal cycler system (Biometra Goettingen, Germany). The PCR procedure included initial denaturation at 94°C for 5 minutes, followed by 30 amplification cycles, each consisting of denaturation at 94°C for 30 seconds, annealing at 56°C for 45 seconds, and extension at 72°C for 45 seconds, with an additional extension step at the end of the procedure at 72°C for 7 minutes. Samples of the PCR products were run on 1% agarose gel with ethidium bromide. The final amount of RT-PCR product for each of the mRNAs species was calculated densitometerically using AIDA 2 (Dinco Co., Israel) software. Each sample was run in duplicates and balanced between groups. The results were corrected for the initial dilution and calculated as the intensity of the lane of each transcript over the intensity of the corresponding *β*-actin band and expressed as a mean in arbitrary units (AU).

### 2.5. Statistical analysis

Neurotrophic factor mRNA levels were assessed by three measurements made in each animal, and the means were calculated.

All data were expressed as the mean ± SEM and statistical analyses were performed using one-way analysis of variance (ANOVA).Where significant group effects were detected, Bonferroni test was used to assess significant post hoc differences between individual groups.

## 3. RESULTS

### 3.1. NF mRNA levels

Table 1 summarizes the levels of neurotrophic factors and TrkB mRNA in the frontal cortex, CA1, CA3, DG, hypothalamus, piriform cortex, and olfactory bulb of naïve, pseudotrained, and trained rats.

In the FC, one-way ANOVA revealed a significant difference in BDNF, TrkB, and NGF mRNA levels between the groups (F(2, 33) = 4.2, *P* < .04; F(2, 33) = 4.2, *P* < .04; F(2, 33) = 4.33, *P* < .04, resp.). Post hoc Bonferroni test revealed that the trained group displayed significantly higher BDNF and TrkB levels as compared to pseudotrained rats (*P* < .04; *P* < .03; *P* < .05) and naïve rats (*P* < .02; *P* < .02; *P* < .05).

The trained group also displayed significantly higher NGF mRNA levels as compared to naïve rats (*P* < .05).

In the hippocampal subregions (CA1, CA3, and DG), hypothalamus, piriform cortex, and olfactory bulb, there were no statistically significant differences between groups in BDNF, TrkB, NGF, and NT-3 mRNA levels.

## 4. DISCUSSION

The results of this study revealed that one day after training for an olfactory discrimination learningtask, the frontal lobes of trained rats demonstrate significant increases in the expression of mRNA for BDNF and TrkB as compared to pseudotrained and naïve animals. Frontal cortex upregulation of NGF mRNA was also observed in the trained rats as compared to the naïve group. Other brain areas, including the hippocampal CA1, CA3, and DG subregions, the piriform cortex, and the olfactory bulb showed no such increased expression. These findings complement prior electrophysiological findings regarding this training task reflecting enhanced synaptic release, as evidenced by reduced PPF [14], enhancement of postsynaptic potentials in pyramidal neurons as indicated byenhanced rate of rise of the PSPs [15] and enhanced connectivity as indicated by an increased number of spines along apical dendrites of these neurons [16–18]. These modifications appear three days after olfactory discrimination learning [14, 17, 19] and are subsequent to enhanced neuronal excitability in the cortical pyramidal neurons. No changes in synaptic activity in the hippocampus were found [22]. Twenty-four hours after olfactory learning, baseline synaptic activity, as well as paired-pulse facilitation, was not enhanced, spine density along dendrites of pyramidal neurons was not yet increased [23], and the subunit composition of the NMDA receptor was similar in the hippocampi of olfactory trained rats and their controls [24]. Thus, our findings clearly indicate that while the hippocampus is involved in forming the olfactory discrimination rule learning [22], the cortex is occupied in maintaining the skill after being acquired [14–16].

Our data suggest that olfactory discrimination learning activates endogenous neurotrophin signaling in the FC, providing intrinsic cortical neurons with more neurotrophic support. These plasticity changes modulate cellular modification of neural networks in the CNS [25, 26] and the selection of functional neuronal connections and neuronal circuit reorganization of the cortex, especially BDNF.

Kesslak et al. [27] have reported that rats trained to locate a submerged platform in a water maze displayed elevated levels of BDNF mRNA in the hippocampus, a structure associated with spatial memory, but other cortical and subcortical areas did not show a significant increase in BDNF mRNA [27]. Hall et al. [28] demonstrate rapid and selective induction of BDNF expression in the CA1 region of the hippocampus during hippocampus-dependent contextual learning. In monkeys, following formation of a declarative memory (pair-association task), Tokuyama et al. [29] showed that BDNF was upregulated selectively in area 36 of inferior temporal cortex, but not in areas involved in earlier stages of visual processing. There are also correlations between hippocampal BDNF mRNA expression and memory performance in senescent rats [30]. Moreover, Broad et al. [31] have reported that 4.5 hours postpartum, the formation of a recognition memory for a lamb was associated with an increased BDNF mRNA expression in the inferior part of the temporal cortex, subfield CA1 of the hippocampus, the diagonal band, basolateral amygdale, and the anterior cingulate, medial frontal, entorhinal, and pyriform cortices. No increases were observed in either the olfactory bulbs or the dentate gyrus [31].

The enhanced levels of NFs, especially BDNF, in the FC are thus in keeping with electrophysiological findings following olfactory learning, but were not found in the area generally associated with learning and memory—the hippocampus. The question is thus whether all learning and memory necessarily occurs only in one area or system, or whether different forms of learning tasks are dealt with or encoded in different brain areas/systems.

In conclusion, we show here that an olfactory discrimination learning task activates production of endogenous NFs concomitant with the induction of signal transduction in the FC, but not in other brain areas. These findings suggest that different brain areas may be preferentially involved in different learning/memory tasks.

## Figures and Tables

**Figure 2:**
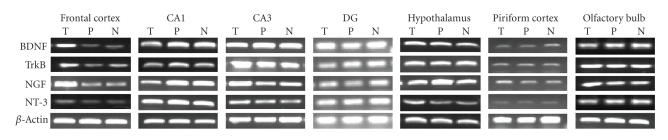
*NF mRNA expression in representative gels*. T, trained; P, pseudotrained; N, naïve.

**Table 1:** *Neurotrophic factors and TrkB mRNA levels expression*/*β-actin in different brain regions amongst groups.* mRNA levels are expressed as mean ± SEM (*n* = 12 in each group) arbitrary unit of each neurotrophic factor or TrkB relative to mRNA levels of *β*-actin. Each sample was run in duplicates.

Neurotrophic factors	Brain region	Trained rats mean ± SEM	Pseudotrained rats mean ± SEM	naïve rats mean ± SEM

BDNF	FC	35.1 ± 4.6* ^#^	9.9 ± 3.7	5.1 ± 0.9
TrkB		23.5 ± 5.6* ^#^	12.9 ± 1.7	9.6 ± 2.2
NGF		18.8 ± 7.4*	9.2 ± 2.9	6.38 ± 2.2
NT-3		1.02 ± 0.35	0.9 ± 0.5	0.75 ± 0.3

BDNF	Hippocampus	9.0 ± 2.4	15.1 ± 5.2	11.2 ± 3.0
TrkB	CA1	4.0 ± 1.1	8.7 ± 2.3	4.45 ± 1.2
NGF		3.35 ± 1.0	2.7 ± 0.6	2.9 ± 0.6
NT-3		1.2 ± 0.5	2.4 ± 0.9	1.3 ± 0.3

BDNF	Hippocampus	11.4 ± 3.9	14.7 ± 4.8	11.1 ± 3.1
TrkB	CA3	3.8 ± 1.1	4.1 ± 2.3	2.8 ± 1.2
NGF		3.1 ± 1.0	2.46 ± 0.7	4.1 ± 1.1
NT-3		3.0 ± 0.9	1.3 ± 0.3	1.5 ± 0.5

BDNF	Hippocampus	7.9 ± 2.2	10.6 ± 3.1	7.0 ± 3.2
TrkB	DG	8.3 ± 0.5	7.8 ± 0.8	3.0 ± 0.7
NGF		5.7 ± 2.2	6.3 ± 2.1	3.2 ± 0.65
NT-3		3.9 ± 1.4	1.3 ± 0.7	0.8 ± 0.4

BDNF	Hypothalamus	12.29 ± 0.7	15.7 ± 1.8	11.0 ± 1.6
TrkB		7.5 ± 1.3	9.37 ± 1.2	9.0 ± 2.0
NGF		4.5 ± 0.9	6.2 ± 1.7	4.3 ± 0.8
NT-3		0.3 ± 0.01	0.5 ± 0.1	0.3 ± 0.02

BDNF	Piriform	8.6 ± 1.7	7.8 ± 1.4	4.3 ± 1.0
TrkB	cortex	4.4 ± 0.9	5.4 ± 0.4	4.1 ± 0.03
NGF		2.8 ± 0.5	2.2 ± 0.5	2.4 ± 1.5
NT-3		0.4 ± 0.1	0.2 ± 0.01	0.1 ± 0.04

BDNF	Olfactory	10.0 ± 2.1	12.2 ± 3.1	9.1 ± 0.5
TrkB	bulb	15.9 ± 1.6	26.1 ± 6.7	17.3 ± 1.8
NGF		10.0 ± 2.1	12.2 ± 3.1	9.1 ± 0.5
NT-3		15.9 ± 1.6	26.1 ± 6.7	17.3 ± 1.8

*P < .05, naïve group versus trained group.

^#^ *P* < .05, pseudotrained group versus trained group.
